# Supplementary material for: Infant and adult human intestinal enteroids are morphologically and functionally distinct
Source: mBio. 2024 Jul 2;15(8):e01316-24. doi: 10.1128/mbio.01316-24 (PMC11323560; doi:10.1128/mbio.01316-24)
Supplement: Figure S1 — MUC2 expression is higher in infant intestinal tissues than adult tissues. [file mbio.01316-24-s0001.pdf]

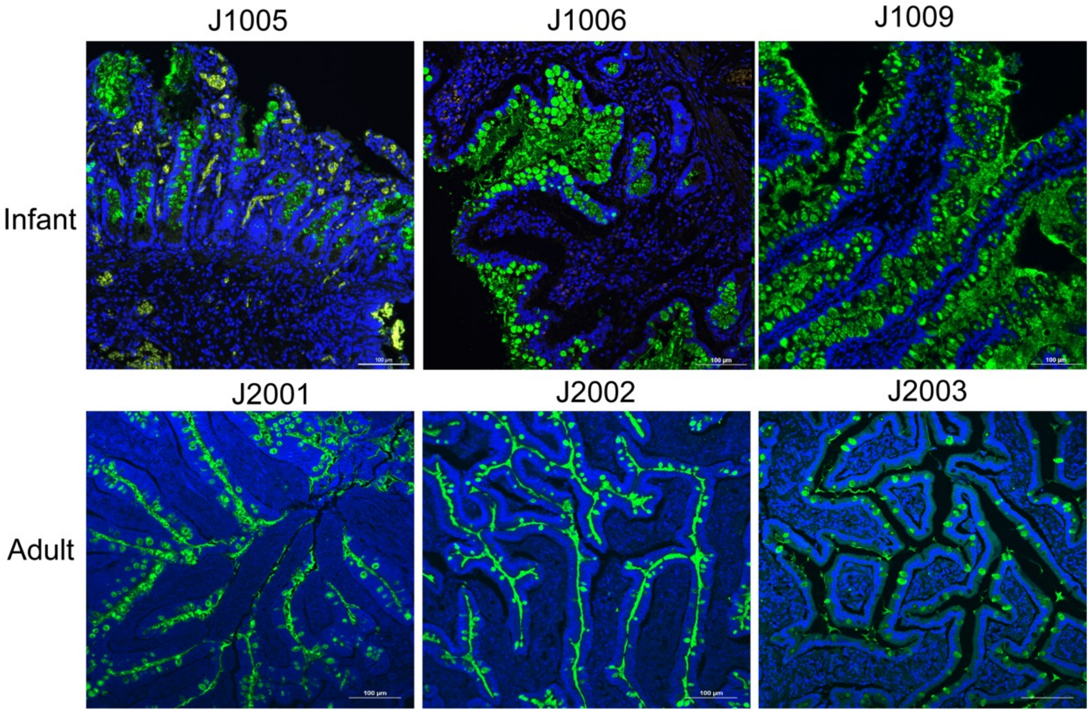

Supplemental Figure 1: MUC2 expression is higher in infant intestinal tissues than adult tissues
Representative confocal images of goblet cells (Muc2, green) in infant and adult tissues from two independent experiments. Nuclei were stained with DAPI (blue). Scale bar = 100 μm.
