# Supplementary material for: Infant and adult human intestinal enteroids are morphologically and functionally distinct
Source: mBio. 2024 Jul 2;15(8):e01316-24. doi: 10.1128/mbio.01316-24 (PMC11323560; doi:10.1128/mbio.01316-24)
Supplement: Figure S2 — There are fewer proliferating cells in differentiated infant and adult HIE monolayers on transwells. [file mbio.01316-24-s0002.pdf]

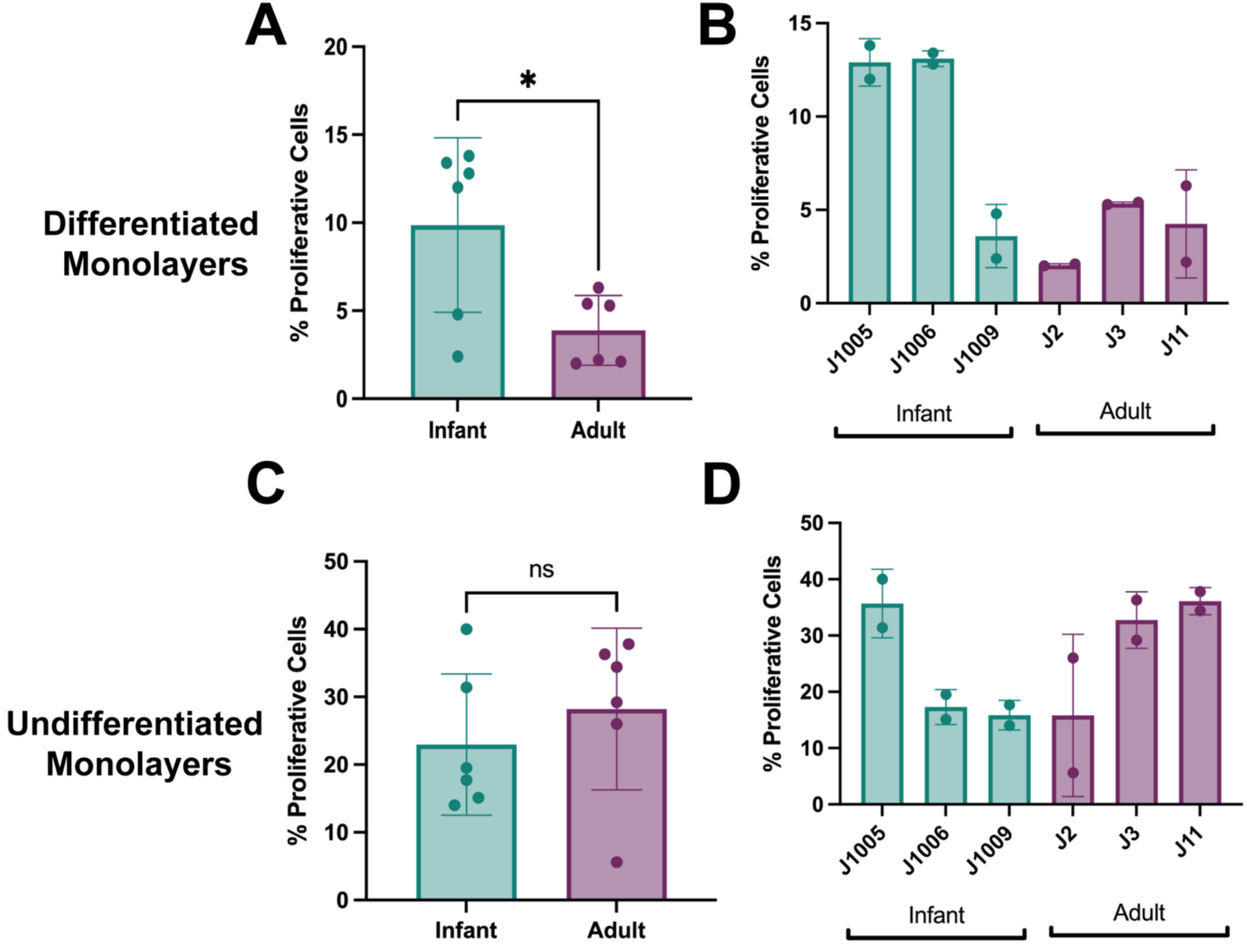

Supplemental Figure 2: There are few proliferating cells in differentiated infant and adult HIE monolayers on transwells

Percentage of EdU-positive cells quantified by flow cytometry in differentiated (A) and undifferentiated (C) HIE monolayers. B&D: Percentage of EdU-positive cells quantified by flow cytometry in individual lines. Data represents mean ± standard deviation (SD) from two independent experiments, with each experiment including the three infant and three adult HIE lines.
