# Supplementary material for: Infant and adult human intestinal enteroids are morphologically and functionally distinct
Source: mBio. 2024 Jul 2;15(8):e01316-24. doi: 10.1128/mbio.01316-24 (PMC11323560; doi:10.1128/mbio.01316-24)
Supplement: Figure S3 — There are few proliferating cells in differentiated infant and adult 3D HIEs. [file mbio.01316-24-s0003.pdf]

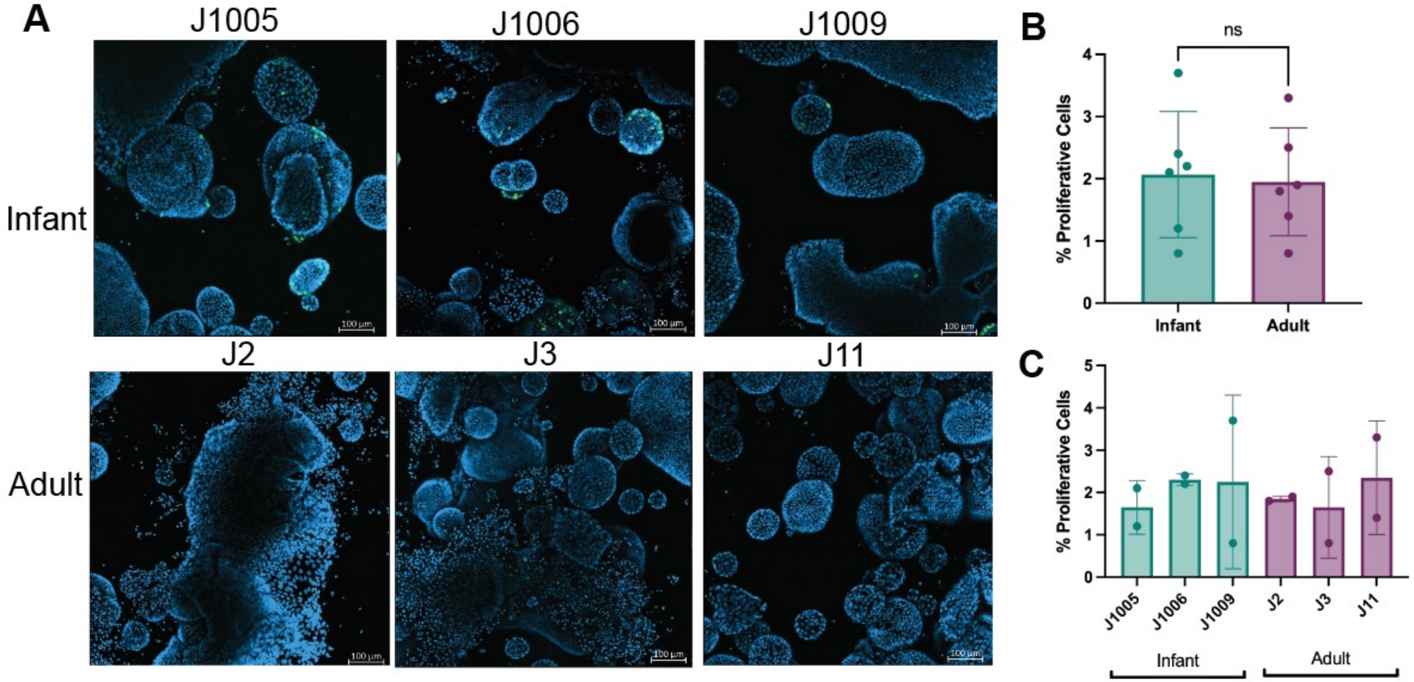

Supplemental Figure 3: There are few proliferating cells in differentiated infant and adult 3D HIEs

A: Representative confocal 3D reconstruction images after 24h 5-ethynyl-2′-deoxyuridine (EdU) incorporation in differentiated infant and adult HIEs. B: Percentage of EdU-positive cells quantified by flow cytometry. C: Percentage of EdU-positive cells quantified by flow cytometry in individual lines. Data represents mean ± SD from two independent experiments, with each experiment including the three infant and three adult HIE lines. The *p*-values were calculated by student's t-test.
