# Supplementary material for: Infant and adult human intestinal enteroids are morphologically and functionally distinct
Source: mBio. 2024 Jul 2;15(8):e01316-24. doi: 10.1128/mbio.01316-24 (PMC11323560; doi:10.1128/mbio.01316-24)
Supplement: Figure S4 — FITC dextran concentration in response to EGTA treatment by line. [file mbio.01316-24-s0004.pdf]

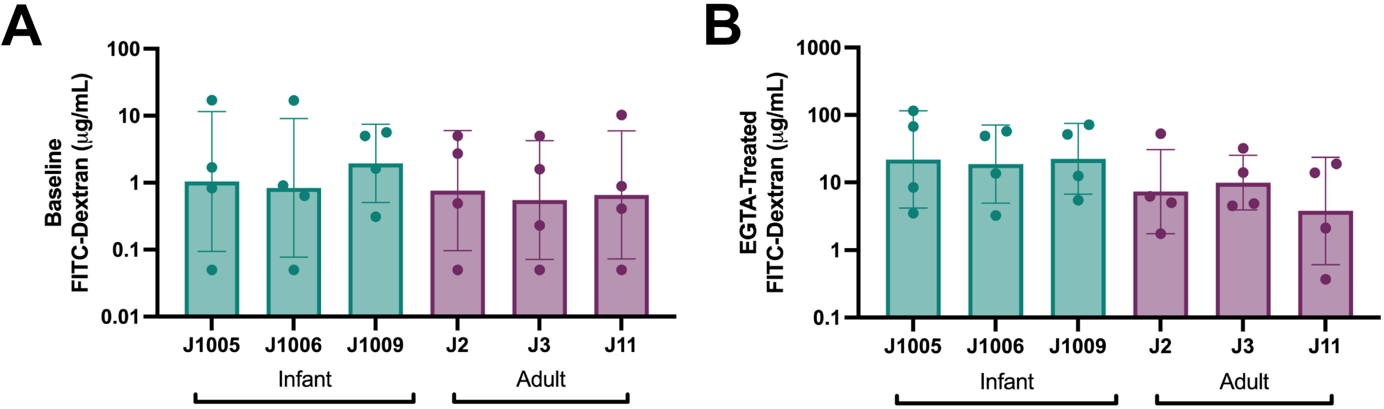

Supplemental Figure 4: FITC-Dextran concentration in response to EGTA treatment by line

Concentration of 4kDA FITC-Dextran in each HIE line at (A) baseline and (B) after EGTA treatment. Data represent mean ± SD from four independent experiments, with each experiment including the three infant and three adult HIE lines.
