# Supplementary material for: Infant and adult human intestinal enteroids are morphologically and functionally distinct
Source: mBio. 2024 Jul 2;15(8):e01316-24. doi: 10.1128/mbio.01316-24 (PMC11323560; doi:10.1128/mbio.01316-24)
Supplement: Figure S5 — TNF-alpha causes changes in TEER in both infant and adult HIEs. [file mbio.01316-24-s0005.pdf]

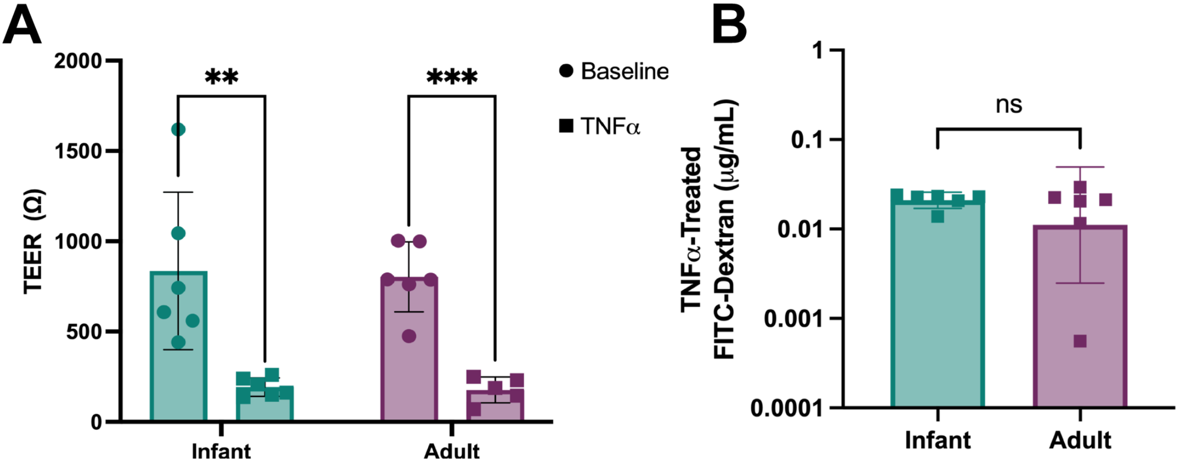

Supplemental Figure 5: TNFα causes changes in TEER in both infant and adult HIEs.

A: TEER values of HIEs at baseline and after TNFα treatment. B: Concentration of 4kDA FITC-Dextran after TNFα treatment. Data represent mean ± SD from two independent experiments, with each experiment including the three infant and three adult HIE lines. The p-values were calculated by student's t-test, and the asterisks (**) and (***) represent $p < 0.01$, and $p < 0.001$ respectively
