# Supplementary material for: Infant and adult human intestinal enteroids are morphologically and functionally distinct
Source: mBio. 2024 Jul 2;15(8):e01316-24. doi: 10.1128/mbio.01316-24 (PMC11323560; doi:10.1128/mbio.01316-24)
Supplement: Figure S6 — HuNoV tissue culture infectious dose 50 and innate immune response to HuNoV infection. [file mbio.01316-24-s0006.pdf]

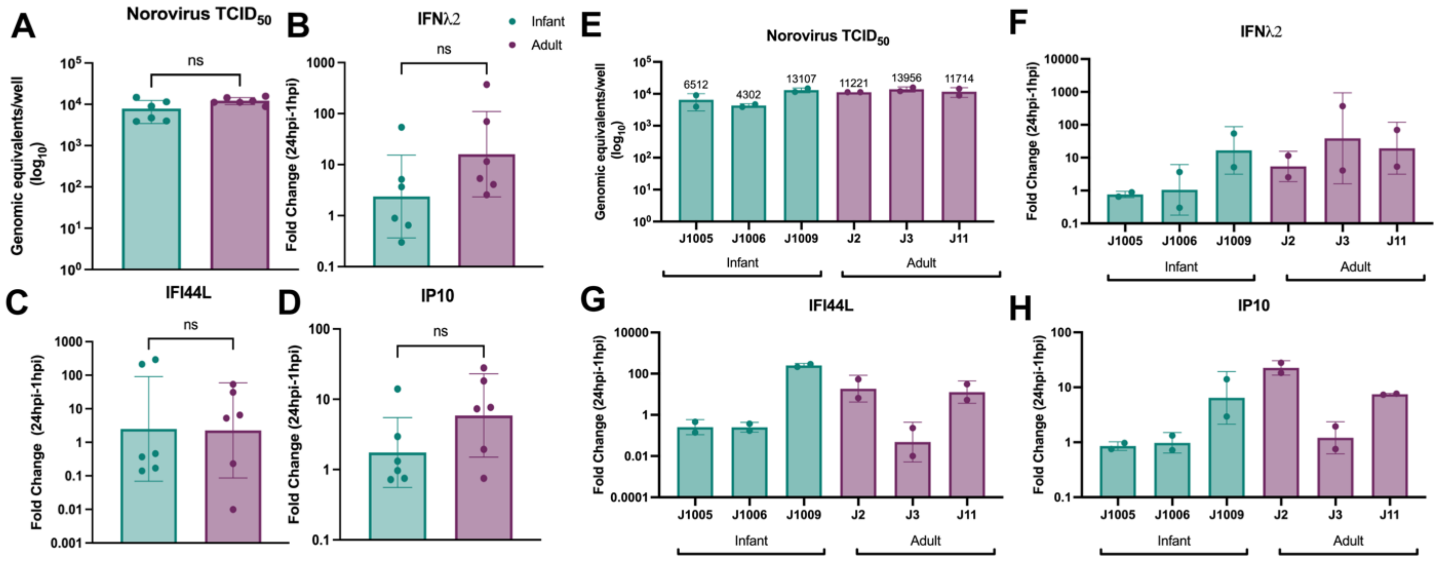


**Supplemental Figure 6: HuNoV tissue culture infectious dose 50 (TCID50) and innate immune response to HuNoV infection**

A: HuNoV (GII.4) tissue culture infectious dose 50 (TCID50) in HIEs at 24hpi. Quantification of (B) IFNλ2, (C) IFI44L, and (D) IP10 transcripts in response to infection with 100 TCID50s of HuNoV. E-H: There are HIE line specific differences in HuNoV TCID50 (E) and innate immune response to HuNoV infection (F-H). Data represent mean ± SD from two independent experiments, with each experiment including the three infant and three adult HIE lines. The p-values were calculated by student's t-test.
