# Supplementary material for: Infant and adult human intestinal enteroids are morphologically and functionally distinct
Source: mBio. 2024 Jul 2;15(8):e01316-24. doi: 10.1128/mbio.01316-24 (PMC11323560; doi:10.1128/mbio.01316-24)
Supplement: Figure S7 — Cell type composition of varies between differentiated 3D infant and adult HIEs. [file mbio.01316-24-s0007.pdf]

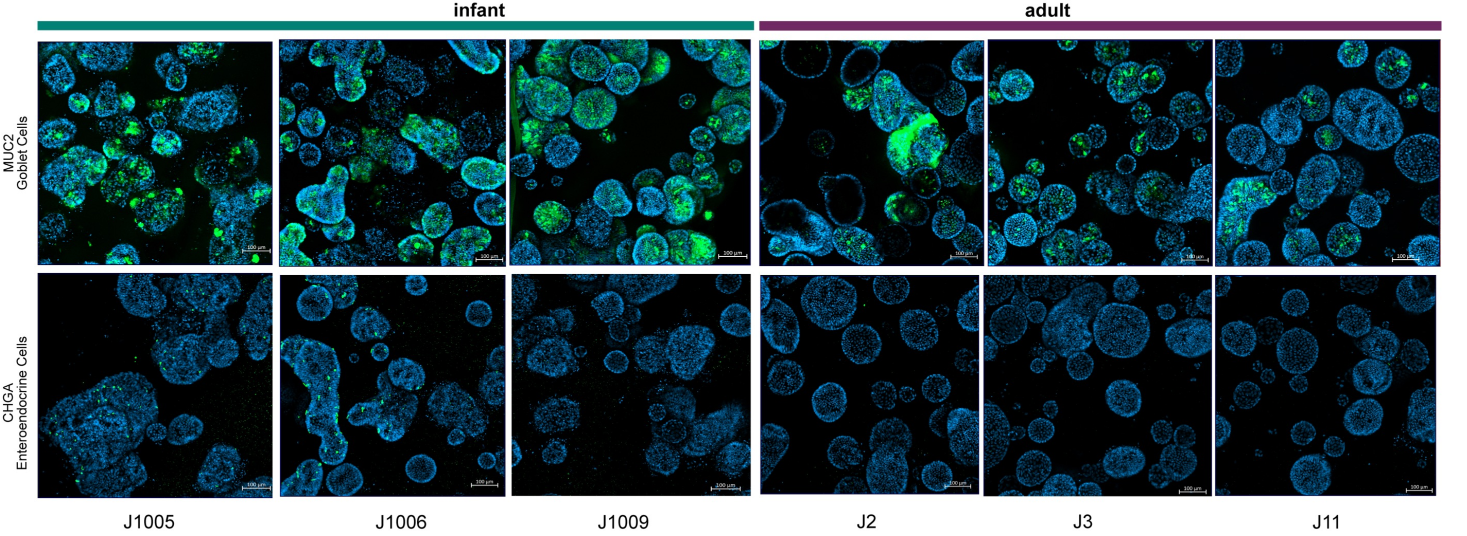

Supplemental Figure 7: Cell type composition of varies between differentiated 3D infant and adult HIEs
A: Representative confocal 3D reconstruction images of differentiated cell types in infant and adult HIEs. Top panel: goblet cells (Muc2, green), and bottom panel: enteroendocrine cells (ChgA, green). Nuclei are stained with DAPI (blue), Scale bar = 100 μm.
