# Supplementary material for: Infant and adult human intestinal enteroids are morphologically and functionally distinct
Source: mBio. 2024 Jul 2;15(8):e01316-24. doi: 10.1128/mbio.01316-24 (PMC11323560; doi:10.1128/mbio.01316-24)
Supplement: Figure S8 — Expression of some toll-like receptors (TLRs) are significantly different in infant HIEs. [file mbio.01316-24-s0008.pdf]

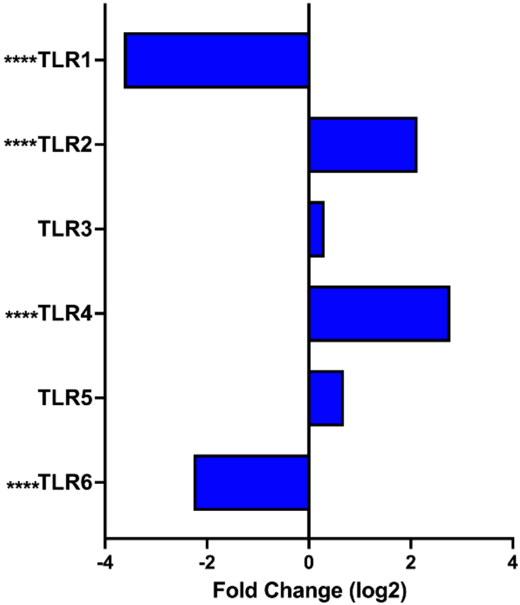

Supplemental Figure 8: Expression of some toll-like receptors (TLRs) are significantly different in infant HIEs

Expression profile of RNA-seq data for TLR genes. Data represents mean values and are expressed as Log2 fold change, the asterisk (****) represents $p < 0.0001$.
