# Supplementary material for: Infant and adult human intestinal enteroids are morphologically and functionally distinct
Source: mBio. 2024 Jul 2;15(8):e01316-24. doi: 10.1128/mbio.01316-24 (PMC11323560; doi:10.1128/mbio.01316-24)
Supplement: Figure S9 — Gating strategy for EdU assay. [file mbio.01316-24-s0009.pdf]

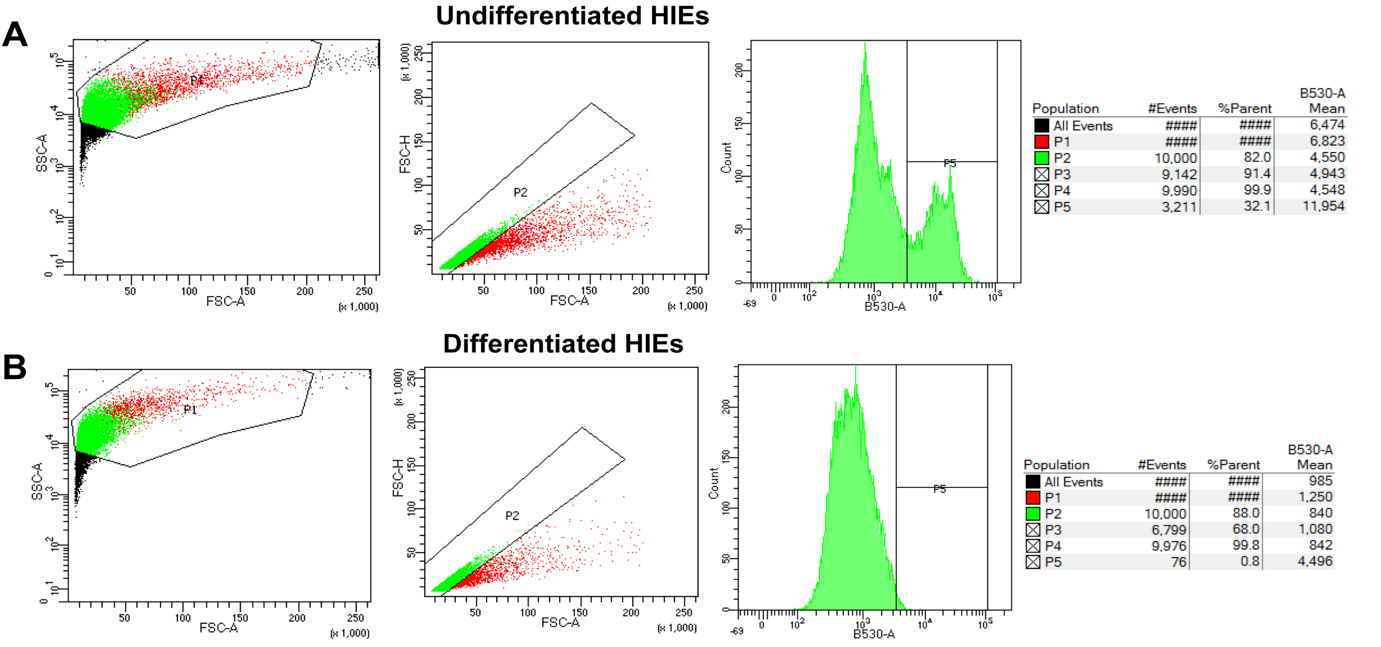

Supplemental Figure 9: Representative EdU gating strategy for undifferentiated (A) and differentiated (B) HIEs
