# Supplementary material for: Infant and adult human intestinal enteroids are morphologically and functionally distinct
Source: mBio. 2024 Jul 2;15(8):e01316-24. doi: 10.1128/mbio.01316-24 (PMC11323560; doi:10.1128/mbio.01316-24)
Supplement: Table S1 — RNA sequencing data on tight junction proteins expressed in infant over adult HIEs and passage number. [file mbio.01316-24-s0010.pdf]

GeneID	GeneSymbol	GeneBiotype	logFC	PValue	FDR
ENSG00000104067	TJP1	Protein coding	0.000	0.999	1.000
ENSG00000119139	TJP2	Protein coding	-0.292	0.511	0.780
ENSG00000105289	TJP3	Protein coding	-0.500	0.046	0.186
ENSG00000197822	OCLN	Protein coding	0.139	0.578	0.827
ENSG00000163347	CLDN1	Protein coding	-1.966	0.000	0.000
ENSG00000165376	CLDN2	Protein coding	4.368	0.000	0.000
ENSG00000165215	CLDN3	Protein coding	0.440	0.129	0.370
ENSG00000189143	CLDN4	Protein coding	-0.200	0.455	0.740
ENSG00000181885	CLDN7	Protein coding	0.122	0.649	0.862
ENSG00000157224	CLDN12	Protein coding	-0.544	0.031	0.140
ENSG00000106404	CLDN15	Protein coding	0.795	0.002	0.015
ENSG00000113946	CLDN16	Protein coding	-1.918	0.003	0.023
ENSG00000066405	CLDN18	Protein coding	-3.358	0.000	0.000
ENSG00000253958	CLDN23	Protein coding	-1.437	0.000	0.000

Supplemental table 1A

RNA Sequencing data on tight junction proteins expressed in infant over adult HIEs.

Main Figure Number	Infant lines	Adult lines
1	5-24	11-21
2	7-12	17-20
3	12-21	5-40
4	2-11	11-28
5	2-45	17-25
6	2-45	6-25
7	10-14	19-22

Supplemental table 1B: Passage number range of HIEs used in primary figures.
